# A chromosome-level reference genome of a Convolvulaceae species *Ipomoea cairica*

**DOI:** 10.1093/g3journal/jkac187

**Published:** 2022-07-27

**Authors:** Fan Jiang, Sen Wang, Hengchao Wang, Anqi Wang, Dong Xu, Hangwei Liu, Boyuan Yang, Lihua Yuan, Lihong Lei, Rong Chen, Weihua Li, Wei Fan

**Affiliations:** Shenzhen Branch, Guangdong Laboratory for Lingnan Modern Agriculture, Genome Analysis Laboratory of the Ministry of Agriculture and Rural Affairs, Agricultural Genomics Institute at Shenzhen, Chinese Academy of Agricultural Sciences, Shenzhen, Guangdong 518120, China; Shenzhen Branch, Guangdong Laboratory for Lingnan Modern Agriculture, Genome Analysis Laboratory of the Ministry of Agriculture and Rural Affairs, Agricultural Genomics Institute at Shenzhen, Chinese Academy of Agricultural Sciences, Shenzhen, Guangdong 518120, China; Shenzhen Branch, Guangdong Laboratory for Lingnan Modern Agriculture, Genome Analysis Laboratory of the Ministry of Agriculture and Rural Affairs, Agricultural Genomics Institute at Shenzhen, Chinese Academy of Agricultural Sciences, Shenzhen, Guangdong 518120, China; Shenzhen Branch, Guangdong Laboratory for Lingnan Modern Agriculture, Genome Analysis Laboratory of the Ministry of Agriculture and Rural Affairs, Agricultural Genomics Institute at Shenzhen, Chinese Academy of Agricultural Sciences, Shenzhen, Guangdong 518120, China; Shenzhen Branch, Guangdong Laboratory for Lingnan Modern Agriculture, Genome Analysis Laboratory of the Ministry of Agriculture and Rural Affairs, Agricultural Genomics Institute at Shenzhen, Chinese Academy of Agricultural Sciences, Shenzhen, Guangdong 518120, China; Shenzhen Branch, Guangdong Laboratory for Lingnan Modern Agriculture, Genome Analysis Laboratory of the Ministry of Agriculture and Rural Affairs, Agricultural Genomics Institute at Shenzhen, Chinese Academy of Agricultural Sciences, Shenzhen, Guangdong 518120, China; Shenzhen Branch, Guangdong Laboratory for Lingnan Modern Agriculture, Genome Analysis Laboratory of the Ministry of Agriculture and Rural Affairs, Agricultural Genomics Institute at Shenzhen, Chinese Academy of Agricultural Sciences, Shenzhen, Guangdong 518120, China; Shenzhen Branch, Guangdong Laboratory for Lingnan Modern Agriculture, Genome Analysis Laboratory of the Ministry of Agriculture and Rural Affairs, Agricultural Genomics Institute at Shenzhen, Chinese Academy of Agricultural Sciences, Shenzhen, Guangdong 518120, China; Shenzhen Branch, Guangdong Laboratory for Lingnan Modern Agriculture, Genome Analysis Laboratory of the Ministry of Agriculture and Rural Affairs, Agricultural Genomics Institute at Shenzhen, Chinese Academy of Agricultural Sciences, Shenzhen, Guangdong 518120, China; Shenzhen Branch, Guangdong Laboratory for Lingnan Modern Agriculture, Genome Analysis Laboratory of the Ministry of Agriculture and Rural Affairs, Agricultural Genomics Institute at Shenzhen, Chinese Academy of Agricultural Sciences, Shenzhen, Guangdong 518120, China; Guangdong Provincial Key Laboratory of Biotechnology for Plant Development, School of Life Science, South China Normal University, Guangzhou 510631, China; Shenzhen Branch, Guangdong Laboratory for Lingnan Modern Agriculture, Genome Analysis Laboratory of the Ministry of Agriculture and Rural Affairs, Agricultural Genomics Institute at Shenzhen, Chinese Academy of Agricultural Sciences, Shenzhen, Guangdong 518120, China

**Keywords:** Ipomoea cairica, Convolvulaceae, chromosome-level assembly, PacBio sequencing, Hi-C sequencing

## Abstract

*Ipomoea cairica* is a perennial creeper that has been widely introduced as a garden ornamental across tropical, subtropical, and temperate regions. Because it grows extremely fast and spreads easily, it has been listed as an invasive species in many countries. Here, we constructed the chromosome-level reference genome of *Ipomoea cairica* by Pacific Biosciences HiFi and Hi-C sequencing, with the assembly size of 733.0 Mb, the contig N50 of 43.8 Mb, the scaffold N50 of 45.7 Mb, and the Benchmarking Universal Single-Copy Orthologs complete rate of 98.0%. Hi-C scaffolding assigned 97.9% of the contigs to 15 pseudo-chromosomes. Telomeric repeat analysis reveals that 7 of the 15 pseudo-chromosomes are gapless and telomere to telomere. The transposable element content of *Ipomoea cairica* is 73.4%, obviously higher than that of other *Ipomoea* species. A total of 38,115 protein-coding genes were predicted, with the Benchmarking Universal Single-Copy Orthologs complete rate of 98.5%, comparable to that of the genome assembly, and 92.6% of genes were functional annotated. In addition, we identified 3,039 tRNA genes and 2,403 rRNA genes in the assembled genome. Phylogenetic analysis showed that *Ipomoea cairica* formed a clade with *Ipomoea aquatica*, and they diverged from each other 8.1 million years ago. Through comparative genome analysis, we reconfirmed that a whole genome triplication event occurred specific to Convolvulaceae family and in the ancestor of the genus *Ipomoea* and *Cuscuta*. This high-quality reference genome of *Ipomoea cairica* will greatly facilitate the studies on the molecular mechanisms of its rapid growth and invasiveness.

## Introduction

*Ipomoea cairica* (L.) Sweet (Convolvulaceae), commonly known as 5-fingered morning glory, is a sprawling and perennial liana with flowers all year around and has been widely introduced as a garden ornamental across tropical, subtropical, and temperate regions, but its exact area of origin is uncertain (Austin and Huaman 1996; Lin *et al.* 2008). The plant grows extremely fast, spreads easily by stem fragments, and has strong adaptive abilities to diverse habitats (Liu *et al.* 2012; 2016). Once naturalized, it has the potential to outcompete native plants, completely invading the space by climbing and covering other plant species in scenic spots, parks, and wild lands, and until now has been listed as an invasive species in many countries, such as Japan, China, Mexico, Australia, and Brazil (Mito and Uesugi 2004; Bai *et al.* 2013; Liu *et al.* 2016). On the other hand, *I. cairica* has medicinal properties because it contains a large number of bioactive compounds, a decoction of the whole plant is used in the treatment of tuberculosis, sough, asthma, liver cirrhosis, and jaundice in many countries (Lima and Braz-Filho 1997; Ma *et al.* 2002; Sumayya *et al.* 2011; Meira *et al.* 2012). Pharmacological activity research has revealed that the extract of the plant has powerful cathartic, larvicidal, anti-inflammatory, anti-nociceptive, and anticancer activities (Ferreira *et al.* 2006; Lin *et al.* 2008; Zuharah *et al.* 2016, 2018).

The genus *Ipomoea*, which consists of 600–700 species, is the largest genus in family Convolvulaceae (Austin and Huaman 1996). Sweet potato (*I. batatas*) is the only species of the genus that is widely cultivated and consumed as a staple crop worldwide. Because the genome of sweet potato is hexaploid (2*n* = 6*x* = 90) and highly polymorphic, it is hard to generate a high-quality reference genome for this species. Although much progress has been made in genome assembly recently, the latest genome assembly of sweet potato is still fragmented, with the scaffold N50 size of only ∼201 kb (Yang *et al.* 2017). To assist in the analysis of the sweet potato genome, the genomes of its 2 diploid relatives (*I*pomoea *trifida* and *Ipomoea triloba*) were also assembled to the chromosome level (Wu *et al.* 2018; Li *et al.* 2019). However, these 2 genome assemblies were still fragmented (with the longest contig N50 size of 65.8 kb), due to the constrains in the sequencing and assembly technologies (Wu *et al.* 2018). In addition, a chromosome-level genome assembly of *Ipomoea aquatica* was generated recently, which made a great improvement in assembly continuity with contig N50 sizes of 1.7 Mb (Hao *et al.* 2021), but still far from some recently published genomes (Liu *et al.* 2022; Lu *et al.* 2022). In this study, we present a high-quality chromosome-level genome for diploid species of *I. cairica* (2*n* = 2*x* = 30) by using Pacific Biosciences (PacBio) HiFi and Hi-C sequencing data. This high-quality genome assembly will greatly facilitate the studies on its molecular mechanisms of the rapid growth and strong adaptive abilities to diverse habitats.

## Materials and methods

### Plant materials’ preparation

**Fig. 1.**
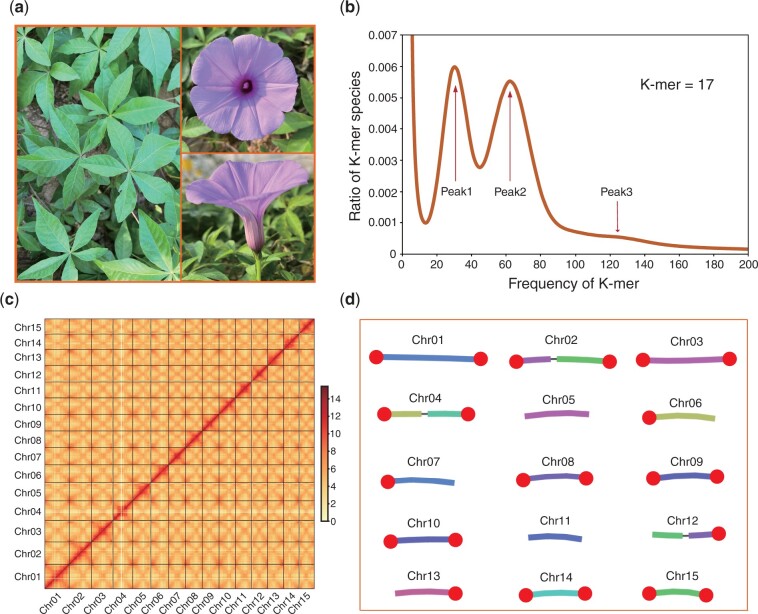
Assessment of the genome assembly for *I. cairica*. a) The photos show the shape of the leaves and flowers for the sequenced *I. cairica*. b) Distribution of K-mer frequencies in sequencing reads. The K-mer frequency peak 1 reflects the “heterozygous” regions, peak 2 reflects the “unique” regions, and peak 3 reflects the “repeats” regions in the genome. K-size equal 17; c) Hi-C heatmap of the genome assembly. We scanned the genome by 1-Mb nonoverlapping window as a bin and calculated valid interaction links of Hi-C data between any pair of bins, and color represents Log2(links number); d) view of the pseudo-chromosomes. The thick lines represent contigs, and the thin lines represent the links between the 2 contigs. The chromosome ends assembled with telomere-specific repeats (AAACCCT) were highlighted with solid circle.

Rhizomes from 1 *I. cairica* (Fig. 1a) individual were collected on roadside near Agricultural Genomics Institute at Shenzhen (latitude 22°35′N, longitude 114°29′E, elevation 33.4 m above sea level), Guangdong, China, in October 2020, and then were cut into approximately 10-cm-long fragments with at least 2 nodes on each fragment. The rhizome cuttings were grown in plastic containers (30 cm × 40 cm) filled with sand in a greenhouse with natural-lit experimental condition, and watered when needed. Three weeks after sprouting, the regenerated plantlets of *I. cairica* were transplanted into pots (diameter 16–20 cm, height 20 cm) filled with mixed growth medium (pond mud:sand:humus = 1:1:1) in the same greenhouse with the same condition.

### DNA extraction and sequencing

Four weeks after transplanting, genomic DNA in young leaves of *I. cairica* was extracted using the Hi-DNAsecure Plant Kit (cat. no. DP350; TIANGEN, China). The integrity of DNA extracts was checked on 0.8% (w/v) agarose gel with GelRed nucleic acid gel stain (cat. no. 41003; Biotium, USA). The purity and quantity of DNA samples were assessed using Nanodrop 2000 (Thermo Fisher Scientific, USA) and Qubit 4.0 (Thermo Fisher Scientific, USA). The DNA samples with high integrity (with obvious concentrated electrophoresis band >15 kb in size) and quality (A260/280 1.8-2.0, dsDNA concentration >50 ng/μl) were used for sequencing.

For Illumina sequencing, a PCR-Free DNA library with 350-bp inserts was constructed using Illumina TruSeq DNA PCR-Free Library Preparation Kit (Illumina, USA) and paired-end sequenced (2 × 150 bp) on an Illumina NovaSeq 6000 platform (Illumina), which produced a total of 57.2 Gb of Illumina reads (Table 1). For PacBio Hifi sequencing, 2 libraries with 15-kb inserts were constructed by SMRTbell Express Template Prep Kit 2.0 (PacBio, USA) and sequenced on a PacBio Sequel II system using circular consensus sequence (CCS) mode (PacBio). CCS reads were generated by ccs v3.0.0 (https://www.pacb.com/support/software-downloads) with parameter “-min-rq 0.99,” and the total size of CCS reads was 53.8 Gb (Table 1). Both Illumina and PacBio sequencing were performed by Nextomics Bioscience Co., Ltd (Wuhan, China). Hi-C experiments were performed as described by Belton *et al.* (2012) using young leaves. The cross-linked DNA was digested with MboI enzyme, and paired-end sequenced (2 × 150 bp) on an Illumina NovaSeq 6000 platform by Annoroad Gene Technology (Beijing, China), generating a total of 89.9 Gb of Hi-C reads (Table 1).

**Table 1.** Summary of the genomic sequencing data for *I. cairica*.

Type	Sequencing platform	Read number	Base number (Gb)	Read length (bp)	Sequencing depth (x)
PacBio	PacBio Sequel II	4,249,439	53.9	13,000 (N50)	74
Illumina	Illumina NovaSeq 6000	190,729,545	57.2	150	78
Hi-C	Illumina NovaSeq 6000	299,790,699	89.9	150	123

### Full-length transcript sequencing

Total RNA was extracted by RNeasy Plant Mini Kit (QIAGEN, Germany) from the root, stem, and leaf and pooled in equal amount and reverse-transcribed into cDNA using NEBNext Single Cell/Low Input cDNA Synthesis & Amplification Module (NEB, UK). The 0.5- to 6-kb cDNA fragments were prepared into sequencing libraries by SMRTbell Express Template Prep Kit 2.0 and sequenced with Iso-Seq mode on PacBio Sequel II system by Nextomics Bioscience Co., Ltd (Wuhan, China). Then, the raw Iso-Seq reads were processed using the IsoSeq3 pipeline to obtain full-length, nonchimeric sequences, and a total of 67,306 full-length transcripts were generated.

### Genome assembly and quality assessment

The genome size and heterozygosity of *I. cairica* were estimated by GCE v1.02 (https://github.com/fanagislab/GCE) (Liu *et al.* 2013) using PacBio HiFi reads. Two SMRT cells of PacBio HiFi reads (a total of 53.9 Gb) were assembled using Hifiasm v0.14.2 (Cheng *et al.* 2021) with parameter “-l 3.” To remove contaminations, the contigs were aligned to the prokaryotic reference genomes and mitochondrion and plastid genomes from NCBI by Minimap2 v2.20 (Li 2018), and the contigs with identity >0.95 and coverage >0.95 were removed. This resulted in 78 contigs with a total length of 733.0 Mb and a contig N50 size of 43.8 Mb (Table 2).

**Table 2.** Statistics of the genomic assembly for *I. cairica*.

Genome assembly	Contigs	Scaffolds
Total length (bp)	733,042,748	733,045,748
Total number	78	75
Maximum (bp)	65,792,717	65,792,717
Minimum (bp)	50,179	50,179
N50 (bp)	43,753,511	45,705,626
N60 (bp)	42,974,940	44,359,965
N70 (bp)	41,122,154	42,974,940
N80 (bp)	36,554,351	42,688,284
N90 (bp)	23,457,119	41,122,154
BUSCO complete rate (%)	98.0	98.0

The quality of the genome assembly was assessed by aligning short-read DNA sequences (Illumina reads) and full-length transcript sequences using BWA-MEM (Li and Durbin 2009) and GMAP v2020-10-27 (Wu and Watanabe 2005), respectively. In addition, yak QV was used to evaluate contig correctness by using Illumina reads (https://github.com/lh3/yak). The Benchmarking Universal Single-Copy Orthologs v5.2.2 (BUSCO) (Simao *et al.* 2015) was also used to evaluate the assembly by testing for the presence and completeness of the orthologs using embryophyta_odb10 database.

### Genome scaffolding

The pseudo-chromosomes were constructed using Hi-C sequencing data. A total of 70.9-Gb Hi-C paired-end clean reads were mapped onto the assembled contigs by Bowtie2 v2.3.4.3 (Langmead and Salzberg 2012), and then HiC-Pro v2.11.4 (Servant *et al.* 2015) pipeline was used to detect valid ligation pairs and generate the Hi-C link matrixes among different contigs. Then, the contigs were clustered, ordered, and oriented into pseudo-chromosomes using EndHiC v1.0 (https://github.com/fanagislab/EndHiC) (Wang *et al.* 2021) based on the Hi-C linkage information among contig ends.

### Repetitive sequence identification

A comprehensive transposable element (TE) analysis was performed for the species *I. cairica*. First, EDTA v1.9.9 (Ou *et al.* 2019) was used to produce a filtered TE library for the annotation of structurally intact and fragmented elements. Second, the TE repeats were identified by homology searching against the above structural TE library, Repbase v26.05 (Bao *et al.* 2015), and protein-coding TE database using RepeatMasker v4.1.2 (Smit *et al.* 2015), which identified 498.5 Mb of TE repeats. Third, an extra de novo TE library was constructed by RepeatModeler v2.0.2 (Flynn *et al.* 2020) from the genome with all identified TE (498.5 Mb) masked, and the unknown TEs in the library were further classified by TERL (da Cruz *et al.* 2021). All classified TE sequences in the de novo TE library were used by RepeatMasker to identify the remaining TEs in the genome. As a result, we found an extra number of 123,644 TE sequences, with the total length of 39,376,506 bp and the average length of 318 bp. In addition, the tandem repeat (TR) elements were investigated using Tandem Repeats Finder (TRF) v4.07 (Benson 1999) with parameter “2 5 7 80 10 50 2000 -h -d.”

### Gene prediction and functional annotation

The TE-masked genome was used for protein-coding gene prediction by using Augustus v3.4.0 (Stanke *et al.* 2006), with transcript and homology hints and parameter “–softmasking=on.” The full-length transcript hints were generated by mapping RNA sequences from PacBio Iso-Seq sequencing to the genome with GMAP v2020-10-27 (Wu and Watanabe 2005), removing the hints with identity <0.95 or coverage <0.95, and transforming to hints by blat2hints.pl in Augustus. The homology hints were generated by aligning the protein-coding sequences from the published genome for the species of *I. aquatica* (Hao *et al.* 2021), *Ipomoea nil* (Hoshino *et al.* 2016), *I. trifida*, and *I. triloba* (Wu *et al.* 2018) to the genome assembly, using Exonerate v2.2.0 (Slater and Birney 2005) with parameter “–% 70,” and transforming to hints by exonerate2hints.pl in Augustus. The training parameters of Augustus were generated during the BUSCO (Simao *et al.* 2015) completeness assessment of the assembled contigs with parameter “–augustus” and applied here. For the gene functional annotation, we aligned the protein sequences of genes to NCBI-NR and KEGG databases using DIAMOND v2 (Buchfink *et al.* 2021) with the *E*-value cutoff of 1e−5, choosing the best hit from the alignment results. The protein domain annotation was performed using InterProScan v5.52-86 (Blum *et al.* 2021) against InterPro database. In addition, the tRNA and rRNA genes were predicted by tRNAscan-SE v2.0 (Chan *et al.* 2021) and RNAmmer v1.2 (Lagesen *et al.* 2007), respectively.

### Evolutionary analysis

The genome data of *I. cairica*, 9 well-assembled species in order Solanales [*I. aquatica* (Hao *et al.* 2021), *I. nil* (Hoshino *et al.* 2016), *I. trifida*, *I. triloba* (Wu *et al.* 2018), *Cuscuta australis* (Sun *et al.* 2018), *C*uscuta *campestris* (Vogel *et al.* 2018), *Solanum lycopersicum* (Mueller *et al.* 2009), *Solanum tuberosum* (Xu *et al.* 2011; Pham *et al.* 2020), and *Capsicum annuum* (Kim *et al.* 2014)), and *Coffea canephora* (Denoeud *et al.* 2014)], were compared. The orthologous groups (orthogroups) were built for these species using OrthoFinder v2.5.2 (Emms and Kelly 2019) with parameter “-M msa -A mafft -T fasttree -l -y.” To infer the phylogeny relationship of these species, the protein sequences of single-copy orthogroups were separately aligned using MUSCLE v3.8.1551 (Edgar 2004) and then concatenated into 1 super sequence for each species. RAxML-NG v1.0.3 (Kozlov *et al.* 2019) was used to build Maximum Likelihood phylogenetic trees with the LG + G8 + F model. The species of *C. canephora* were used as outgroup for the phylogeny analysis. The divergence time was estimated using MCMCtree within the package PAML v4.10.0 (Yang 2007), setting the calibration time of 79–91 million years ago (MYA) between *C*. *canephora* and Solanales species, which was obtained from the website of TimeTree (www.timetree.org). Subsequently, the expansion and contraction of the gene families relative to its ancestors were estimated using CAFE v5.0 (Mendes *et al.* 2021) with parameter “-k 3.”

To investigate the whole genome triplication (WGT) event occurred in the evolutionary history of *Ipomoea* species, collinear blocks of inter- and intraspecies for the genome of *I. cairica*, *I. aquatica*, *I. nil*, *I. trifida*, *I. triloba*, *C. australis*, and *S. lycopersicum* were determined using MCScanX (Wang *et al.* 2012) from the alignment files generated during the orthogroup construction. The java programs of circle_plotter and dot_plotter inside the MCScanX were used to draw the genome-wide synteny figures. Then, the distributions of pairwise synonymous rates (*Ks*) of paralogous genes from collinear blocks were calculated. The collinear blocks with more than 5 syntenic gene pairs were used for the *Ks* distribution analysis, and *Ks* values were calculated using KaKs_calculator (Wang *et al.* 2010) with the GMYN model.

## Results

### Chromosome-level genome assembly of *I. cairica*

To obtain a high-quality genome, 53.8 Gb (73×) of PacBio HiFi reads were generated with a read N50 length of 13 kb (Table 1). Prior to genome assembly, the genome size of the *I. cairica* was estimated to be 730 Mb based on k-mer frequencies (Fig. 1b), with a heterozygosity rate of 1.02%. Then, these reads were used to assemble a reference genome by Hifiasm, followed by filtering of the short contaminated contigs. The assembled genome includes 78 contigs with a total length of 733.0 Mb (Table 2), which is comparable to the estimated genome size. The contigs N50 and N90 sizes of the genome assembly are 43.8 and 23.5 Mb (Table 2), respectively, which are much longer than that of other published *Ipomoea* species (Supplementary Table 1). The accuracy of the genome assembly was assessed by mapping Illumina short-read DNA sequences and full-length transcripts to the genome, which revealed that 97.1% and 99.9% of the DNA sequences and transcripts, respectively, could be aligned to the genome assembly. In addition, the quality of the final assembly was estimated to be QV40 (accuracy 99.99%) by using Illumina reads, suggesting that our genome assembly is of high accuracy. Then, the completeness of the genome assembly was evaluated using BUSCO (Simao *et al.* 2015) based on the embryophyta_odb10 database, revealing a complete rate of 98.0% for the genome assembly of *I. cairica* (Table 2 and Supplementary Table 1).

With Hi-C technology, 717.4 Mb (97.9%) of contigs were successfully anchored to 15 pseudo-chromosomes (Fig. 1c, Table 3, and Supplementary Fig. 1), which corresponded to the 15 chromosomes of the species (Dutta 2017). Of the 15 pseudo-chromosomes, 12 contains only 1 contig and 3 contains 2 contigs (Fig. 1d and Table 3). The value of GC content for all pseudo-chromosomes is similar, and the average value is 36.4% (Table 3), which is consistent to that of *I. aquatica* (35.1%), *I. nil* (37.0%), *I. trifida* (35.3%) and *I. triloba* (35.6%) (Hoshino *et al.* 2016; Wu *et al.* 2018; Hao *et al.* 2021). In addition, the telomeric repeat units (AAACCCT) were identified based on the result from TRF (Benson 1999), which showed that 73.3% of the assembled chromosome ends have telomeric repeats, and 9 pseudo-chromosomes were found to have telomeric repeats at both the ends, and 4 pseudo-chromosomes had telomeric repeats at 1 end (Fig. 1d). In summary, we obtained a nearly complete high-quality chromosome-level reference genome for *I. cairica* with the N50 and N90 sizes of 45.7 and 41.1 Mb, respectively (Table 2), and 7 of the 15 pseudo-chromosomes were gapless and Telomere-to-Telomere (Fig. 1d).

**Table 3.** Statistics of the pseudo-chromosomes.

ID	Length (bp)	Contig no.	Gaps (bp)	G + C (%)
Chr01	65,792,717	1	0	36.61
Chr02	58,719,702	2	1,000	35.81
Chr03	57,176,273	1	0	36.61
Chr04	51,515,914	2	1,000	37.23
Chr05	48,098,784	1	0	36.11
Chr06	47,573,477	1	0	36.77
Chr07	45,705,626	1	0	36.35
Chr08	44,607,093	1	0	36.17
Chr09	44,359,965	1	0	36.19
Chr10	43,753,511	1	0	36.43
Chr11	42,974,940	1	0	36.99
Chr12	42,688,284	2	1,000	35.88
Chr13	42,619,119	1	0	36.64
Chr14	41,122,154	1	0	36.42
Chr15	40,670,372	1	0	36.04
Total	717,377,931	18	3,000	36.42

Gaps were preset as 1,000 Ns.

### Higher proportion of repeat elements

In total, the *I. cairica* genome comprises 73.4% (537.9 Mb) of nonredundant TE repeats (Table 4 and Supplementary Fig. 1**)**, including 60.7 Mb of structural intact TEs. The most predominant TE elements are long terminal repeats (48.7%) and DNA transposon elements (21.9%) (Table 4), which account for about 96.1% of the total TE elements. Compared to other *Ipomoea* species of *I. aquatica* (54.8%), *I. nil* (63.3%), *I. trifida* (50.2%), and *I. triloba* (52.8%), obviously higher proportion of TE repeats is found in *I. cairica* (73.4%) (Supplementary Table 2), which may result from the higher continuity of the reference genome (Supplementary Table 1) and a more comprehensive TE identification method for *I. cairica*. The most abundant components of TE repeats in *I. cairica* are Gypsy (21.9%) and Copia (10.7%), which are consistent with that of *I. aquatica* (Supplementary Table 2). In addition, the TR elements were also investigated using TRF (Benson 1999), and we identified a total of 74.8 Mb (10.20%) TRs in *I. cairica* genome (Supplementary Fig. 1), with an N50 size of 1,152 bp for the TR sequences.

**Table 4.** Statistics of transposable element content in various classes.

TE class	Length (bp)	% of genome
LTR	356,815,754	48.7
DNA elements	160,314,758	21.9
MITE	9,774,250	1.3
LINE	9,331,501	1.3
SINE	1,252,236	0.2
RC	392,761	0.1
Others	2,129	0.0
Total	537,883,389	73.4

LTR, long terminal repeat; MITE, miniature inverted-repeat transposable element; LINE, long interspersed nuclear element; SINE, short interspersed element; RC, rolling-circle transposable element.

### Gene prediction and annotation

We predicted a total of 38,115 (42.16 Mb) protein-coding gene models by using Augustus (Stanke *et al.* 2006), with an average coding sequence (CDS) length of 1,106 bp, a mean exon number of 4.7, and a BUSCO (Simao *et al.* 2015) complete rate of 98.5% by using embryophyta_odb10 database, comparable to that of other published *Ipomoea* species (Table 5). In addition, the complete rate of the predicted genes was consistent with that of the assembled genome sequences (98.0%) (Table 2). For gene function annotation, 78.4%, 59.8%, and 91.4% of genes were annotated by NCBI-NR, KEGG, and InterPro database, respectively, and a total of 92.6% of genes could be functionally annotated by at least one of the above databases. In addition, we identified 3,039 tRNA genes and 2,403 rRNA genes in the assembled genome.

**Table 5.** Comparison of gene set between *I. cairica* and other *Ipomoea* species.

Gene prediction	*I. cairica*	*I. aquatica*	*I. nil*	*I. trifida*	*I. triloba*
Gene number	38,115	29,606	35,151	32,301	31,426
Average exon number	4.68	5.17	4.90	4.95	5.03
Average exon length (bp)	236	233	273	248	248
Total exon length (bp)	42,156,936	35,698,410	47,058,378	39,785,558	39,374,739
Average CDS length (bp)	1,106	1,205	1,338	1,231	1,252
BUSCO assessment (%)					
Complete	98.5	95.9	99.3	95.6	96.6
Complete and single copy	93.3	88.8	94.2	90.6	92.1
Complete and duplication	5.2	7.1	5.1	5.0	4.5
Fragmented	0.9	2.2	0.1	2.0	1.4
Missing	0.6	1.9	0.6	2.4	2.0

### Phylogenetic analysis and divergence time estimation

**Fig. 2.**
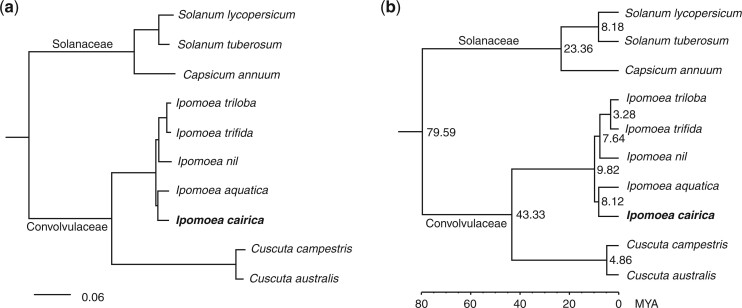
Genome evolution analysis for *I. cairica*. a) Phylogeny tree constructed by RAxML using concatenated protein sequences from 391 single-copy genes. The outgroup species of *C. canephora* was not shown. The bar means substitution per amino acid site; b) the divergence time was estimated by MCMCtree within the package PAML, and setting the calibration time of 79–91 MYA between *C*. *canephora* and Solanales species. The node labels indicate estimated divergence time.

To explore the relationships among *I. cairica* and other related species, gene sets from 9 Solanales species (*I. aquatica*, *I. nil*, *I. trifida*, *I. triloba*, *C. australis*, *C. campestris*, *S. lycopersicum*, *S. tuberosum*, and *C. annuum*) and *C. canephora* were analyzed. A total of 339,245 genes were clustered into 28,248 orthogroups (with each orthogroup containing at least 2 genes), of which 391 were single-copy orthogroups. Then, we constructed a maximum-likelihood tree based on sequence information from these 391 single-copy orthogroups. The topology of the resulted phylogenetic tree showed that *I. cairica* formed a clade with *I. aquatica* in family Convolvulaceae (Fig. 2a), which was consistent with the species tree built by OrthoFinder (Supplementary Fig. 2) using 2,883 orthogroups with a minimum of 90.9% of species having single-copy genes in any orthogroup, confirming the accuracy of the phylogenetic relationships among these species.

**Fig. 3.**
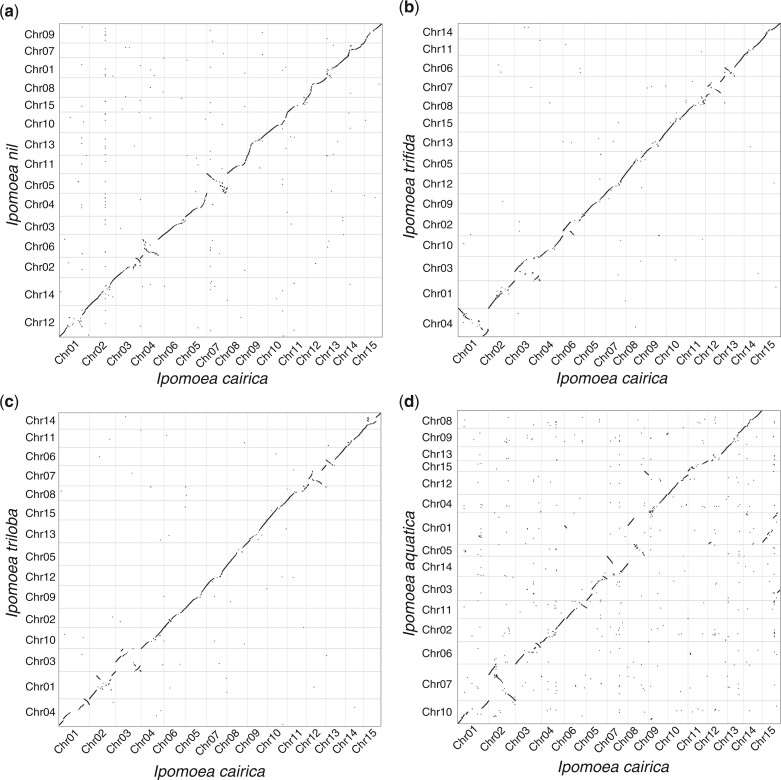
Comparisons between the genomes of *I. cairica* and other *Ipomoea* species. Pair-wise alignment of genome sequences between *I. cairica* and a) *I. nil*, b) *I. trifida*, c) *I. triloba*, and d) *I. aquatica* that were performed using Minimap2 with parameter “-x asm5.”

The divergence time on the phylogenetic tree was estimated by MCMCtree within the package PAML (Yang 2007). The result showed that *I. cairica* and *I. aquatica* diverged from each other 8.1 MYA and they diverged from the other *Ipomoea* species 9.8 MYA (Fig. 2b), which was close to a previous study that indicated *I. aquatica* diverged from the other *Ipomoea* lineage 7.1 (5.4–9.7) MYA (Hao *et al.* 2021). Comparisons between the genomes of *I. cairica* and other *Ipomoea* species were performed using Minimap2 (Li 2018). The results showed an obvious one-to-one syntenic relationships for all 15 chromosomes between *I. cairica* and *I. nil*, *I. trifida*, and *I. triloba* (Fig. 3, a–c), suggesting that limited large-scale interchromosomal rearrangements had occurred after their divergences. However, more interchromosomal rearrangements were observed between the genomes of *I. cairica* and *I. aquatica* (Fig. 3d), though these 2 species were much closer in the phylogenic relationship (Fig. 2a), possibly due to the errors in the pseudo-chromosome assembly for *I. aquatica*. In addition, the expansion and contraction of the gene families relative to its ancestors were estimated using CAFE (Mendes *et al.* 2021), which showed that 1,302 gene families were expanded and 1,320 gene families were contracted in *I. cairica* (Supplementary Fig. 3).

### Reconfirmation of a WGT event occurred for the *Ipomoea* lineage

A previous study that sequenced the genome of *I. nil* reported a whole genome duplication (WGD) event occurred independently in the Convolvulaceae family (Hoshino *et al.* 2016). However, later studies based on the genome sequence of *I. trifida* and *I. triloba* indicated a WGT event occurred in the *Ipomoea* genome instead of the reported WGD (Wu *et al.* 2018; Li *et al.* 2019).

**Fig. 4.**
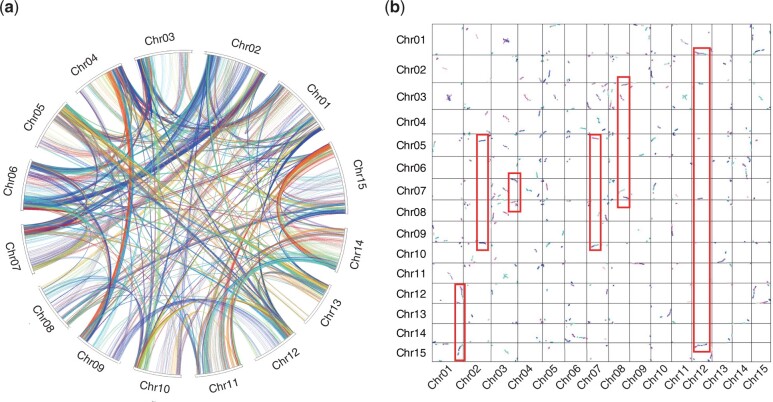
Circle (a) and dot (b) figures showing the intraspecies chromosome synteny for the genome of *I. cairica*. The collinear fragments with more than 10 syntenic gene pairs were plotted, and some examples showing the triples formed by the WGT event in the *Ipomoea* ancestor were highlighted with rectangular.

**Fig. 5.**
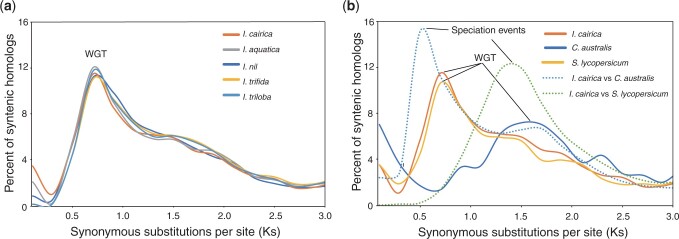
Ks distribution of orthologous or paralogous genes for *I. cairica* and related species. a) Distributions of *Ks* within genomes of *I. cairica*, *I. aquatica*, *I. nil*, *I. trifida*, and *I. triloba*. b) Distributions of *Ks* within genomes *I. cairica*, *C. australis*, and *S. lycopersicum* were showed with solid lines and between genomes of *I. cairica* and the related *C. australis* and *S. lycopersicum* were showed with the line of dashes.

To study the conservation of genomic structure, we identified 12,079 (31.7%) intraspecies syntenic genes by MCScanX (Wang *et al.* 2012) within *I. cairica*, and visualization of the intraspecies synteny indicated that some genome fragments were present in triplicate (Fig. 4). Then, we calculated the Ks values of the paralog pairs in the syntenic fragments for each species. The *Ks* distributions within *I. cairica* and other 4 *Ipomoea* species showed similar peaks at ∼0.7 (Fig. 5a), which were consistent with a previous study that reported *Ks* peaks at 0.65 for *I. trifida* and *I. triloba* (Wu *et al.* 2018), confirming that a recent whole-genome polyploidization event occurred in *Ipomoea* species (Hoshino *et al.* 2016; Wu *et al.* 2018; Li *et al.* 2019; Hao *et al.* 2021). Based on the above results, we reconfirmed a WGT event instead of WGD event that occurred in an ancestor of the *Ipomoea* lineage (Wu *et al.* 2018; Li *et al.* 2019). The *Ks* distributions among the species of *I. cairica*, *C. australis*, and *S. lycopersicum* showed that the *Ks* peak at 0.7 from syntenic paralogs of *I. cairica* occurred after the speciation peak at 1.4 between *I. cairica* and *S. lycopersicum* and before the speciation peak at 0.5 between *I. cairica* and *C. australis* (Fig. 5b), which were consistent with a previous study that analyzed using 4DTv data (Hao *et al.* 2021), reconfirming that the WGT event occurred specific to Convolvulaceae family and in the ancestor of the genera *Ipomoea* and *Cuscuta* (Sato *et al.* 2012; Sun *et al.* 2018; Wu *et al.* 2018; Li *et al.* 2019).

## Discussion

In this study, we utilize the accurate long reads of PacBio HiFi sequencing technology and generate a highly contiguous genome assembly for species *I. cairica*, which has the longest contig N50 size (43.8 Mb) among the published genomes of the genus *Ipomoea*. Phylogenetic analysis indicated that *I. cairica* was closely related to *I. aquatica*, and they diverged from their common ancestor about 8.1 MYA. Through comparative genomics analysis, we reconfirmed a WGT event instead of WGD event occurred in an ancestor of the *Ipomoea* lineage. This high-quality genome assembly will greatly facilitate the studies on the molecular mechanisms of the rapid growth and invasiveness of *I. cairica*.

Sweet potato (*Ipomoea batatas*), the seventh most important crop in the world, is the only staple crop in genus *Ipomoea* that is widely cultivated and consumed worldwide. Because the genome of sweet potato is hexaploid and highly polymorphic, the published genome assembly of this species is highly fragmental and until now there lacks a highly continuous and accurate reference genome (Yang *et al.* 2017), hindering the investigations of some agronomical traits based on the genetics and genomics studies. To assist the construction of chromosome-level genome for sweet potato, the genome assembly of a diploid species *Ipomoea nil* related to sweet potato was used as a reference (Yang *et al.* 2017), but the resulted assembly was still in low quality (Wu *et al.* 2018). Here, a much higher contiguous genome assembly was generated for another related diploid *Ipomoea* species (*I. cairica*), which may improve the genome assembly of sweet potato when used as a reference sequence. In addition, *I. cairica* possesses the characteristics of rapid growth, strong capacity for vegetative propagation, and strong adaptive abilities to diverse habitats. Studies on the key genes underlying these traits may provide some cues for improving the agronomic traits of sweet potato by molecular breeding methods.

## Data availability

All raw sequencing data generated during the current study have been deposited at DDBJ/ENA/GenBank under project accession PRJNA820303. Genomic sequence reads have been deposited in the SRA database with accession SRR18493763 and SRR18493762 for PacBio and Illumina sequencing, respectively. Full-length transcript sequence reads have been deposited in the SRA database with accession SRR18493760. Hi-C sequencing reads have been deposited in the SRA database with accession SRR18493761. Genomic assembly, supporting data and materials are available at the AGIS (ftp://ftp.agis.org.cn/~fanwei/Ipomoea_cairica_genome_v1). Data are available at Zenodo: https://doi.org/10.5281/zenodo.6792002.

Supplemental material is available at *G3* online.

## Supplementary Material

jkac187_Supplementary_Data
