# Supplementary material for: A chromosome-level reference genome of a Convolvulaceae species Ipomoea cairica
Source: G3 (Bethesda). 2022 Jul 27;12(9):jkac187. doi: 10.1093/g3journal/jkac187 (PMC9434287; doi:10.1093/g3journal/jkac187)
Supplement: jkac187_Supplementary_Data [file jkac187_supplementary_data.docx]

**Supplementary Information**

**A chromosome-level reference genome of a Convolvulaceae species *Ipomoea cairica***

Fan Jiang, Sen Wang, Hengchao Wang, et. al

**Supplementary figures**

**
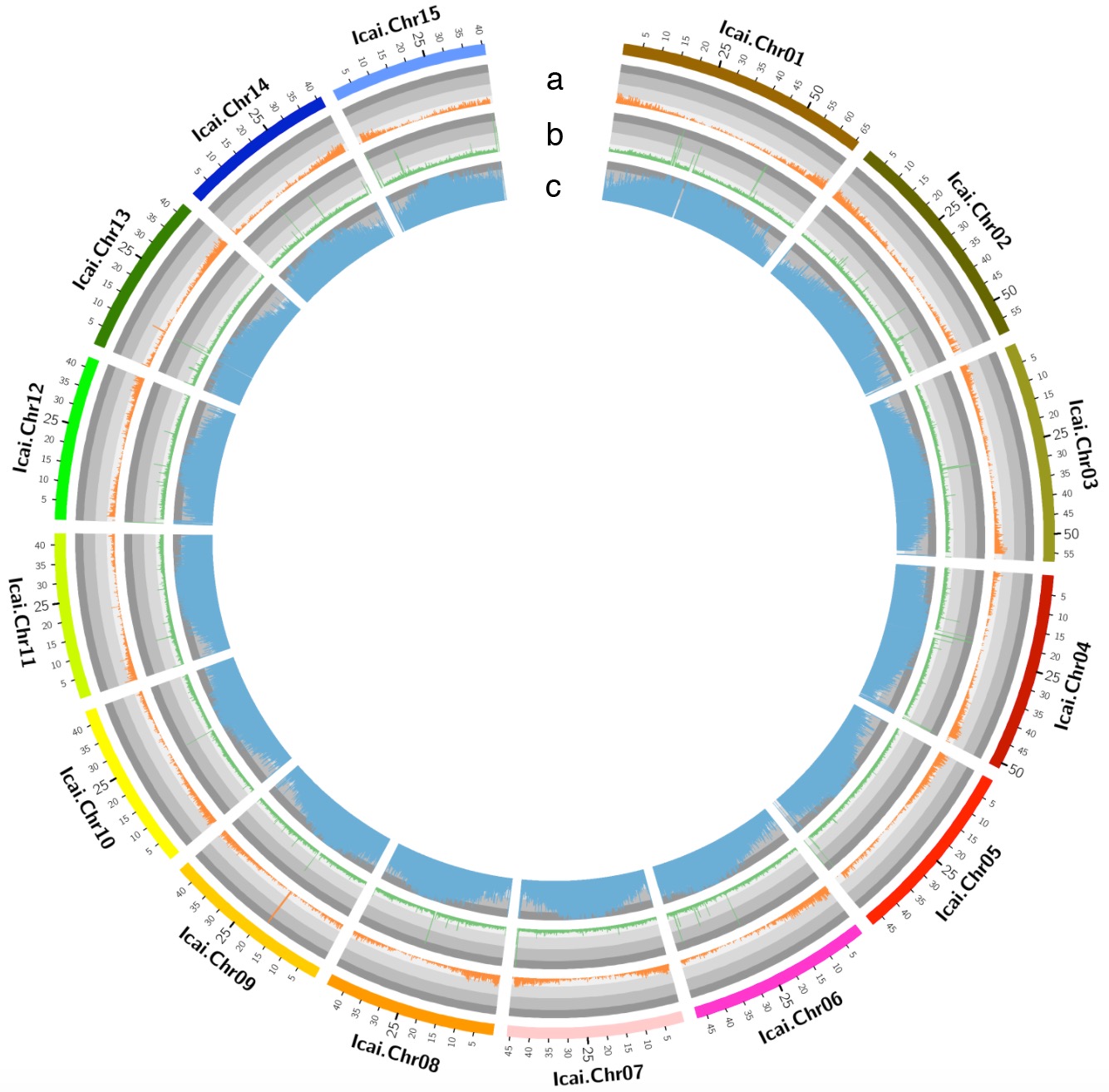
**

**Supplementary Figure S1.** The genome landscape of *Ipomoea cairica*. Circular representation of the chromosomes. Tracks a-c represent the distribution of gene density, tandem repeat density, and transposable element (TE) density, respectively, with densities calculated in 100-kb windows.


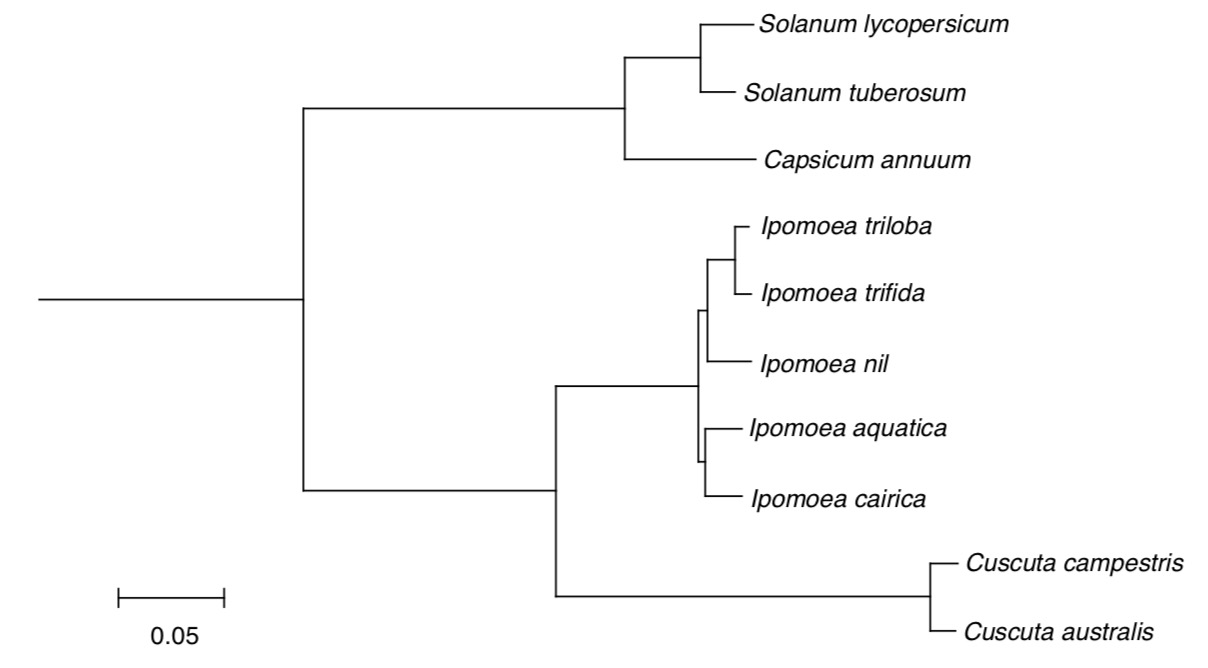


**Supplementary Figure S2.** Species tree of *Ipomoea cairica* and related Solanales species. The species tree constructed by STAG in OrthoFinder, using 2,883 orthogroups with minimum of 90.9% of species having single-copy genes in any orthogroup. Bar means substitution per amino acid site.


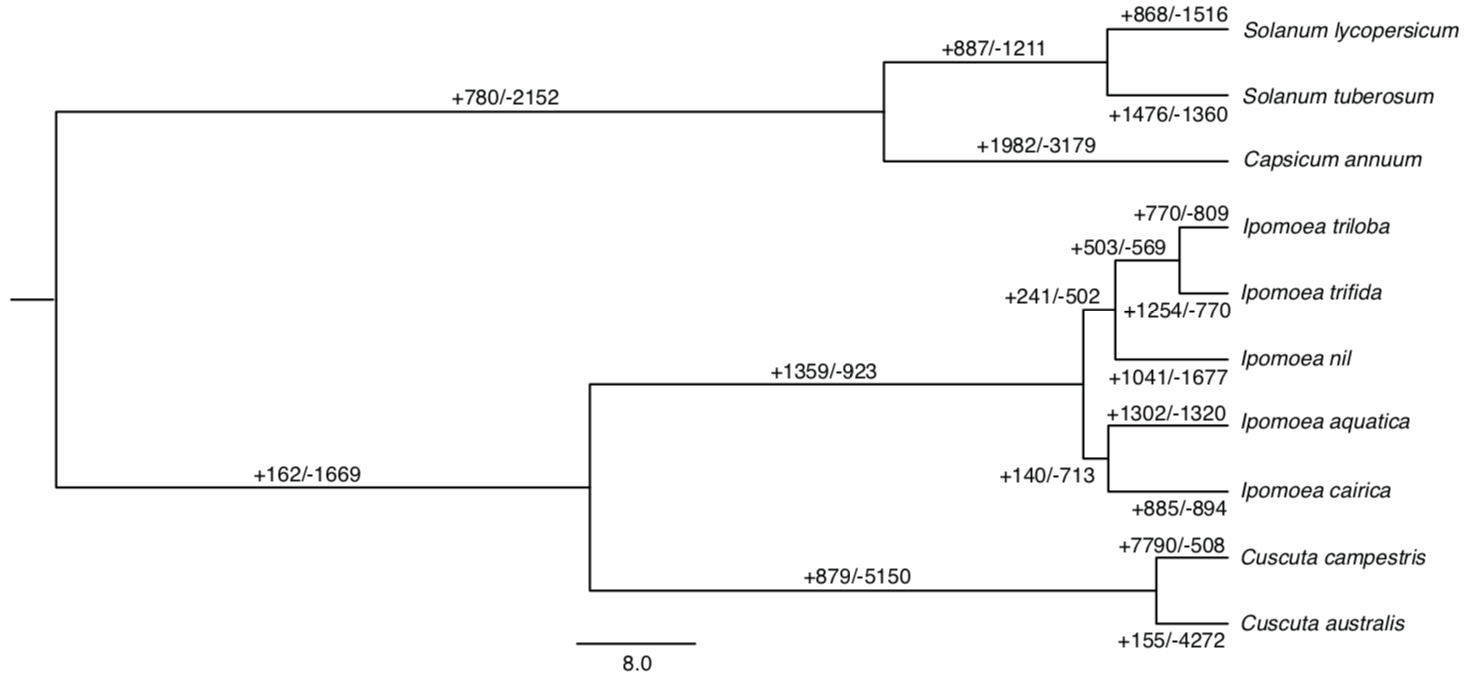


**Supplementary Figure S3.** Gene family (orthogroup) expansion and contraction analysis. The number of expanded (“+”) and contracted (“-”) families for each species were shown on the branch of the species tree.

**Supplementary tables**

**Supplementary Table S1.** Comparisons of genome assembly between *Ipomoea cairica* and other *Ipomoea* species

| **Genome assembly** | *I. cairica* | *I. aquatica* | *I. nil* | *I. trifida* | *I. triloba* |
| --- | --- | --- | --- | --- | --- |
| Contig N50 (bp) | 43,753,511 | 1,650,363 | 1,830,236 | 65,820 | 36,931 |
| Contig N90 (bp) | 23,457,119 | 224,045 | 271,635 | 4,204 | 8,536 |
| Scaffold N50 (bp) | 45,705,626 | 2,753,594 | 3,727,853 | 1,237,020 | 6,861,300 |
| Scaffold N90 (bp) | 41,122,154 | 447,773 | 571,756 | 22,554 | 2,051,804 |
| BUSCO assessment |  |  |  |  |  |
| Complete | 98.0% | 98.1% | 98.1% | 97.8% | 98.3% |
| Complete and single-copy | 93.2% | 91.3% | 93.4% | 92.6% | 93.6% |
| Complete and duplication | 4.8% | 6.8% | 4.7% | 5.2% | 4.7% |
| Fragmented | 0.7% | 0.5% | 0.7% | 0.8% | 0.5% |
| Missing | 1.3% | 1.4% | 1.2% | 1.4% | 1.2% |

**Supplementary Table S2.** Comparisons of transposable element content between *Ipomoea cairica* and other *Ipomoea* species

| **TEs annotation** | *I. cairica* | *I. aquatica* | *I. nil* | *I. trifida* | *I. triloba* |
| --- | --- | --- | --- | --- | --- |
| Total TEs | 73.4% | 54.8% | 63.3% | 50.2% | 52.8% |
| Copia | 10.7% | 10.7% | 12.9% | 6.2% | 8.4% |
| Gypsy | 21.9% | 24.2% | 14.5% | 5.4% | 7.0% |
